# Improving Crop Yield by Regulating Crop Growth and Nitrogen Transformation Through Water and Nitrogen Management Under Subsurface Drip Irrigation System

**DOI:** 10.3390/plants15142171

**Published:** 2026-07-15

**Authors:** Ziye Zhang, Yan Liu, Xin Zhang, Aijun Zhang, Yang Liu, Jing Zhou

**Affiliations:** College of Resources and Environmental Sciences, Hebei Agricultural University, Baoding 071000, China; 15636313980@163.com (Z.Z.); 13869621563@163.com (Y.L.); 15533120289@163.com (Y.L.); 17865279396@163.com (J.Z.)

**Keywords:** subsurface drip irrigation, winter wheat–summer maize rotation, crop yield, N utilization

## Abstract

To address water scarcity, environmental pollution from excessive fertilization, and the need for stable grain yields in the North China Plain (NCP), a 3-year field experiment (2021–2024) was conducted to explore the effects of water and nitrogen (N) management under subsurface drip irrigation on winter wheat–summer maize rotation system. This study included three irrigation treatments (irrigated to 80% (D1), 75% (D2), and 70% (D3) of field water-holding capacity (WHC) when soil water content dropped below 65%, 60%, and 55% of WHC, respectively) under N210 and four N application rates (0 (N0), 150 (N150), 210 (N210), and 270 (N270) kg N ha^−1^ for each season) under D1. The results showed that D1 and N210 all significantly improved leaf area index (LAI) and aboveground biomass by reducing chlorophyllase and pheophytinase activities (delaying leaf senescence) and enhancing nitrate reductase (NR), glutamine synthetase (GS), and glutamate synthase (GOGAT) activities (promoting N assimilation). Furthermore, compared with D3, D1 increased winter wheat and summer maize yields by 12.7% and 24.3%, respectively, and improved partial factor productivity (PFP) by 14.0–46.0%. Redundancy analysis (RDA) and structural equation modeling confirmed that LAI, biomass, and N-transforming enzyme activities were the key drivers of yield. This study demonstrated that the combination of D1 and N210 treatments were the optimal water and N fertilizer management strategies for achieving water conservation, N reduction, and stable high yields in drip-irrigated rotation systems of the NCP.

## 1. Introduction

Globally, irrigation water consumption during agricultural production accounts for over 70% of total anthropogenic water consumption [1]. The North China Plain (NCP) is a key intensive agricultural production region for the wheat–maize rotation system in China, contributing 67% and 28% of national wheat and maize yields, respectively, under conventional irrigation with high fertilizer input rates [2]. Nevertheless, prolonged overexploitation of groundwater for irrigation, coupled with excessive fertilization, has given rise to a series of environmental problems, including soil salinization, waterlogging and nutrient imbalance [3]. Therefore, synergistically achieving water conservation, N reduction, and stable high yield is urgently required for sustainable agriculture in the NCP.

Nitrogen (N) and water are widely regarded as the dominant factors affecting crop growth, N uptake and transformation, which ultimately govern plant N distribution, translocation, utilization, and yield formation [4]. Appropriate N application reduced chlorophyllase activity, delayed leaf senescence and increased the leaf area index (LAI) [5], while also enhancing the activities of nitrate reductase (NR) and glutamine synthetase (GS) to promote N metabolism and improve dry matter translocation [6]. Moderate water stress could enhance root acclimation and drought resistance [7], promote N translocation to grains, and increase grain N content, yield and N use efficiency (NUE) [8]. However, long-term water stress could inhibit root nitrate uptake and N assimilation through decreasing NR and GS activities [9]. Thus, optimizing irrigation and N application is critical for balancing resource conservation and crop productivity.

Drip fertigation enables precise delivery of water and nutrients to the root zone, and has been recognized as an effective strategy for improving N use efficiency, saving water, and maintaining high yields [10]. However, few studies have reported that fertigation could maintain or enhance plant NR, GS, and GOGAT enzyme activities, significantly improved N uptake efficiency [11], and effectively regulated physiological characteristics (such as increasing LAI and SPAD values), thereby promoting dry matter accumulation and ultimately improving N translocation efficiency from leaves to grains [12]. Furthermore, how subsurface drip fertigation enhanced N translocation, yield, and N use efficiency through the modulation of plant physiology and N transformation remained poorly understood. Therefore, this study aimed to (1) investigate the effects of different water and N management regimes on crop growth and physiological traits; (2) clarify their influence on plant N distribution and utilization; and (3) identify the key pathways through which water and N management affect yield, thereby providing technical support for achieving stable yield, water saving, and fertilizer N reduction in the NCP.

## 2. Materials and Methods

### 2.1. Experimental Site

The experiment was conducted in Gaoyang County, Baoding City, Hebei Province (115°38 ′E, 38°46′ N). The region had a typical temperate continental monsoon climate with distinct four seasons. The annual average precipitation was 515.2 mm, with significant interannual fluctuations and obvious seasonal differences. The experimental period was from October 2021 to October 2024, and the changes in daily average temperature and rainfall over this period were presented in Figure S1. The soil at the experimental site was classified as loam. In 0–20 cm soil layer, the pH value was 7.8; the organic matter content was 20.29 g kg^−1^, total N was 0.92 g kg^−1^, available phosphorus was 13.81 mg kg^−1^, and available potassium was 150.23 mg kg^−1^. The overall soil fertility was at a medium level.

### 2.2. Experimental Design

The sowing dates of winter wheat in this experiment were 31 October 2021, 24 October 2022, and 20 October 2023, corresponding to harvest dates of 18 June 2022, 13 June 2023, and 13 June 2024, respectively. The winter wheat variety was Shannong 29, with a sowing rate of 375 kg ha^−1^. Summer maize (Qiule 368, with a density of 60,000 plants ha^−1^) was sown on 30 June 2022, 19 June 2023, and 17 June 2024, and harvested on 25 September 2022, 2 October 2023, and 4 October 2024, respectively. A total of three irrigation regimes and four fertilizer N application rates were established. The irrigation treatments: irrigated to 80% of the field water-holding capacity (WHC) when the soil water content (SWC) was less than 65% of the WHC (D1), irrigated to 75% of WHC when the SWC was less than 60% of the WHC (D2), and irrigated to 70% of WHC when the SWC was less than 55% of the WHC (D3), and all were fertilized with 420 kg N ha^−1^ per year; the fertilization treatments: N0 (no N application), N150 (150 kg N ha^−1^ per season), N210 (210 kg N ha^−1^ per season), and N270 (270 kg N ha^−1^ per season); all were irrigated followed with D1. Each treatment was assigned to a single large plot of 1550 square meters (62 m long × 25 m wide), and three representative sampling zones were designated within each plot to serve as three replicates for soil and plant sampling. The more details for calculation of irrigation water, irrigation and fertilization events were shown in Supporting Information and Figure S1. Accordingly, the total rainfall through winter wheat–summer maize rotation were 542.7 mm (2021–2022), 745.5 mm (2022–2023), and 727.0 mm (2023–2024). The total irrigation water received for D1, D2 and D3 was 167.0 mm, 146.9 mm, and 140.2 mm (2021–2022), 227.9 mm, 191.3 mm, and 150.6 mm (2022–2023), and 234.6 mm, 190.0 mm, and 158.4 mm (2023–2024), respectively.

### 2.3. Plant Sample Collection and Analysis

#### 2.3.1. Determination of Leaf Area Index (LAI), Aboveground Biomass and Plant N Contents

At the jointing, flowering, filling and maturity stages of winter wheat and at the large bell, tasseling, and maturity stages of summer maize, 6 representative plants were selected from each plot to measure the green leaf area, which was calculated using the length–width coefficient method. The leaf area (A) was calculated as: A = L × W × K, where K is 0.75 for fully expanded leaves. The green leaf area index (LAI, m^2^ m^–2^) was the total green leaves area to the area occupied by those plants. Then the plant samples were oven-dried at 70 °C in a forced draft oven to constant weight and then weighed to calculate biomass. Plant subsamples were passed through a 1 mm mesh and mineralized using H_2_SO_4_–H_2_O_2_ for the analysis of N contents by the Kjeldahl method using a Kjeltec™ 8400 auto-distillation unit (Foss Tecator, Eden Prairie, MN, USA).

#### 2.3.2. Determination of Plant Enzyme Activity

Fresh leaf samples were ground rapidly with liquid nitrogen. The powder was homogenized in pre-cooled extraction buffer and kept on ice throughout the operation. The homogenate was centrifuged at 12,000 rpm for 20 min at 4 °C, and the supernatant was collected as crude enzyme extract for subsequent assays.

(1) Chlorophyllase and pheophytinase (PPH)

The reaction system was configured strictly according to the referenced method. The reaction was initiated under constant temperature and terminated after a fixed incubation time. The absorbance was determined spectrophotometrically at 667 nm.

(2) Nitrate reductase (NR)

The reaction mixture contained substrate solution and crude enzyme liquid. The reaction proceeded at 25 °C in the dark and was then terminated. The absorbance was measured at 540 nm.

(3) Glutamine synthetase (GS) and Glutamate synthase (GOGAT)

The enzymatic reaction was carried out at 37 °C. After the reaction was terminated, the absorbance of GS was read at 540 nm, and the absorbance of GOGAT was determined at 340 nm. One unit (U) was defined as the amount of enzyme that caused a 0.01 change in absorbance per minute. All enzyme activities were expressed as U g^−1^. The enzyme activity was calculated with the formula:

(1) $$Enzyme\;activity=\frac{∆A\times V_{t}}{FW\times V_{s}\times t}$$

where ∆*A* is the absorbance change derived from the standard curve (µmol), *V_t_* is the total volume of the enzyme extract (mL), *FW* is the fresh weight of the sample (g), *V_s_* is the volume of enzyme solution used in the assay (mL), and *t* is the incubation time (min).

### 2.4. Yield, N Translocation and Utilization

At the maturity stage of wheat, a 1 m^2^ sample plot with uniform growth was selected within each plot for yield measurement. The number of ears per unit area was determined by counting the ears in 1 m^2^, and 20 ears were randomly selected to record the number of grains per ear. The wheat plants from the 1 m^2^ sample plot were threshed and weighed to calculate the yield per unit area (kg ha^−1^). And 1000 grains were randomly counted, dried and weighed, repeated 3 times, and the 1000-grain weight and yield were calculated according to 13% water content. At the maturity stage of maize, a 5 m double row with uniform growth was selected from each plot for yield measurement. The number of effective ears was counted to calculate the number of ears per unit area. After harvest, 10 ears were selected from each plot for threshing. After drying the grains, the yield per unit area was converted according to 14% water content. A total of 500 grains were randomly counted and weighed, and the 100-grain weight was calculated according to 14% water content, repeated 3 times. 

Partial factor productivity (PFP):

(2) $$\mathrm{PFP}=\frac{Y}{N_{ap}}$$

where *PFP* is the partial factor productivity of N fertilizer (kg kg^−1^), *Y* is the grain yield (kg ha^−1^), and *N_ap_* is the N application rate (kg ha^−1^).

Contribution rate of N translocation from vegetative organs to grains (NTPC):

(3) $$N_{\mathrm{trans}}=N_{\mathrm{flower}}-N_{\mathrm{residual}}$$

(4) $$NTPC=\frac{N_{trans}}{N_{grain}}\times 100\%$$

where NTPC is the contribution rate of N translocation from vegetative organs to grains, *N_flower_* is the total N uptake of plants at the flowering stage, *N_residual_* is the residual N uptake of vegetative organs (stems, leaves) at the maturity stage, and *N_grain_* is the total N uptake in grains at the maturity stage.

Contribution rate of dry matter translocation from vegetative organs to grains (BTPC):

(5) $$W_{\mathrm{trans}}=W_{\mathrm{flower}}-W_{\mathrm{residual}}$$

(6) $$BTPC=\frac{W_{trans}}{{Yield}_{grain}}\times 100\%$$

where *W_flower_* is the total aboveground dry weight at the flowering stage, *W_residual_* is the residual aboveground dry weight at the maturity stage, and *Yield_grain_* is the total grain yield (dry weight) at the maturity stage.

### 2.5. Data Statistics and Analysis

All experimental data were collated and preprocessed using Excel 2010 to remove abnormal values and ensure data validity. Origin 9.0 software (OriginLab Corporation, Northampton, MA, USA) was employed for generating figures (e.g., dynamic curves of growth indicators, radar charts of enzyme activities, scatter plots of yield fitting, and correlation heatmaps) to visualize the data trends and relationships. SPSS 22.0 software (SPSS Inc., Chicago, IL, USA) was used for all statistical tests. The Shapiro–Wilk and Levene tests confirmed that all data were normally distributed and met the assumption of homogeneity of variance. The one-way analysis of variance (ANOVA) was performed to examine the effects of different irrigation (D1, D2, D3) and N application (N0, N150, N210, N270) treatments on crop growth, N content, enzyme activity, yield, and N utilization characteristics. The least significant differences (LSD) test was adopted for multiple comparisons between treatment means, with a significance level set at *p* < 0.05. Correlation analysis (Pearson correlation coefficient) was conducted to clarify the relationships between yield, N utilization parameters (PFP, NTPC, BTPC), plant growth traits (LAI, biomass, plant N contents), and enzyme activities (Chlo, PPH, NR, GS, GOGAT). Redundancy analysis (RDA) and variance partitioning analysis (VPA) were performed using Canoco 5.0 software (Microcomputer Power, Ithaca, NY, USA) to identify the key factors affecting crop yield under different irrigation and N management conditions. Structural equation modeling was established using AMOS 24.0 software (IBM Corp., Armonk, NY, USA) to verify the direct and indirect impact pathways of irrigation and N fertilizer on crop yield, nutrient translocation rate, and N uptake. All statistical results were presented as mean ± standard deviation.

## 3. Results

### 3.1. Plant Growth Characteristics

**Figure 1:**
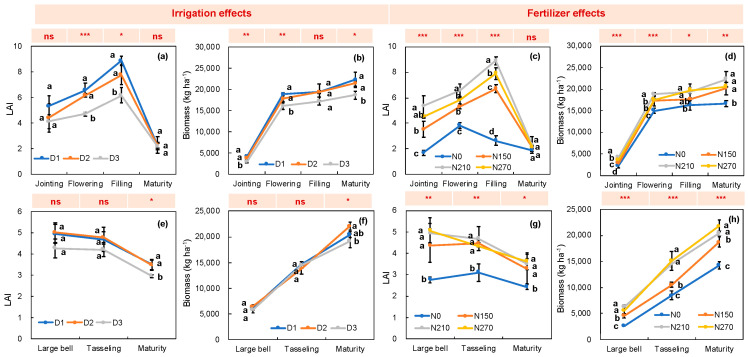
The effects of irrigation and fertilization on leaf area index (LAI) and biomass of winter wheat (**a**–**d**) and summer maize (**e**–**h**) at different stages. Different lowercase letters indicate significant differences in the same growth stage at *p* < 0.05. The symbols of ns, *, **, and *** represent no significant effects and significant effects at 0.05, 0.01 and 0.001 significance levels of irrigation or fertilization on leaf area index (LAI) and biomass of wheat and maize in different growth stages. Definitions of different treatments (i.e., D1, D2, D3. N0, N150, N210 and N270) are given in the caption of Table 1.

Across all treatments, the LAI of winter wheat peaked at the filling stage (Figure 1). Under different irrigation treatments, D1 exhibited the highest LAI, showing a 43.5% increase compared with D3 (*p* < 0.05); under different N application treatments, N210 showed the highest LAI, which was 237.5% (*p* < 0.05) higher than that of N0. The increase in irrigation amount and N application rate both significantly affected biomass. At the maturity stage, the D1 and N210 treatments exhibited the highest biomass, with significant increase of 19.7% and 34.0% compared with D3 and N0, respectively. In summer maize season, the LAI gradually decreased after the large bell stage, while the biomass gradually increased. At the maturity stage, N application and irrigation significantly affected LAI and biomass. Under different irrigation and N application treatments, LAI and biomass were the lowest in D3 and N0 treatments, respectively.

**Table 1:** The yield, N use and transformation of wheat and maize under different irrigation and fertilization treatments.

Treatment	Wheat Season							Maize Season					
	Yield	PFP	N Uptake		NTPC	BTPC		Yield	PFP	N Uptake		NTPC	BTPC
			Flowering	Maturity						Flowering	Maturity		
	Mg ha^−1^	kg kg^−1^	kg N ha^−1^	Kg N ha^−1^	%	%	Mg ha^−1^	kg kg^−1^	kg N ha^−1^		Kg N ha^−1^	%	%
D1	8.5 ± 0.6 a	40.4 ± 2.8 a	363.5 ± 13.1 a	417.8 ± 40.7 a	84.5 ± 6.4 a	69.9 ± 12.5 a	10.3 ± 0.3 a	49.2 ± 1.6 a	322.0 ± 21.1 a		364.1 ± 14.5 a	86.3 ± 6.4 a	59.2 ± 2.3 a
D2	8.3 ± 0.6 a	39.6 ± 2.7 a	322.0 ± 27.7 b	381.1 ± 10.8 a	80.9 ± 9.1 a	65.9 ± 2.4 a	9.5 ± 0.6 a	45.4 ± 2.9 a	225.5 ± 21.9 c		335.8 ± 29.3 a	57.5 ± 7.8 b	37.0 ± 5.5 b
D3	7.5 ± 0.4 a	35.9 ± 1.9 a	217.0 ± 12.2 c	300.3 ± 30.4 b	67.4 ± 11.9 a	71.6 ± 13.2 a	8.3 ± 0.4 b	39.6 ± 1.8 b	282.4 ± 15.5 b		289.7 ± 5.2 b	96.6 ± 5.6 a	59.2 ± 10.1 a
ANOVA	ns	ns	***	**	ns	ns		**	**	**	**	***	*
N0	5.8 ± 0.4 b	/	139.7 ± 20.8 c	175.3 ± 2.4 c	76.1 ± 14.2 a	80.3 ± 14.0 a		6.7 ± 0.3 c	/	153.2 ± 20.9 c	191.6 ± 25.1 c	75.1 ± 14.3 ab	34.7 ± 11.2 b
N150	7.9 ± 0.8 a	52.8 ± 5.1 a	248.1 ± 23.4 b	317.6 ± 20.3 b	72.9 ± 6.2 a	72.0 ± 22.2 a	8.6 ± 0.3 b	57.6 ± 2.1 a	208.4 ± 5.2 b		283.8 ± 13.7 b	63.9 ± 5.1 b	28.3 ± 8.3 b
N210	8.5 ± 0.6 a	40.4 ± 2.8 b	363.5 ± 13.1 a	417.8 ± 40.7 a	84.5 ± 6.4 a	69.9 ± 12.5 a	10.3 ± 0.3 a	49.2 ± 1.6 b	322.0 ± 21.1 a		364.1 ± 14.5 a	86.3 ± 6.4 ab	59.2 ± 2.3 a
N270	8.7 ± 0.4 a	32.2 ± 1.5 c	289.6 ± 34.7 b	354.7 ± 21.5 b	77.6 ± 6.9 a	70.3 ± 1.2 a		10.1 ± 0.4 a	37.3 ± 1.5 c	333.1 ± 42.3 a	337.4 ± 14.1 a	98.7 ± 19.2 a	50.9 ± 18.7 ab
ANOVA	***	***	***	***	ns	ns		***	***	***	***	*	*

Note: Values in the table are presented as mean ± standard deviation (SD). Different lowercase letters within the same column indicate significant differences in the same year at *p* < 0.05. ns, *, **, and *** indicate no significant difference and significant differences at the 0.05, 0.01, and 0.001 probability levels, respectively. D1: irrigated to 80% of the field holding capacity when the soil water content was less than 65% of the field holding capacity; D2: irrigated to 75% of the field holding capacity when the soil water content was less than 60% of the field holding capacity; D3: irrigated to 70% of the field holding capacity when the soil water content was less than 55% of the field holding capacity; N0: no nitrogen application; N150: 150 kg N ha^−1^ per season; N210: 210 kg N ha^−1^ per season; and N270: 270 kg N ha^−1^ per season; NTPC: the contribution rate of N translocation from vegetative organs to grains; BTPC: the contribution rate of dry matter translocation from vegetative organs to grains; PFP: the partial factor productivity of N fertilizer.

### 3.2. Plant N Content Characteristics

**Figure 2:**
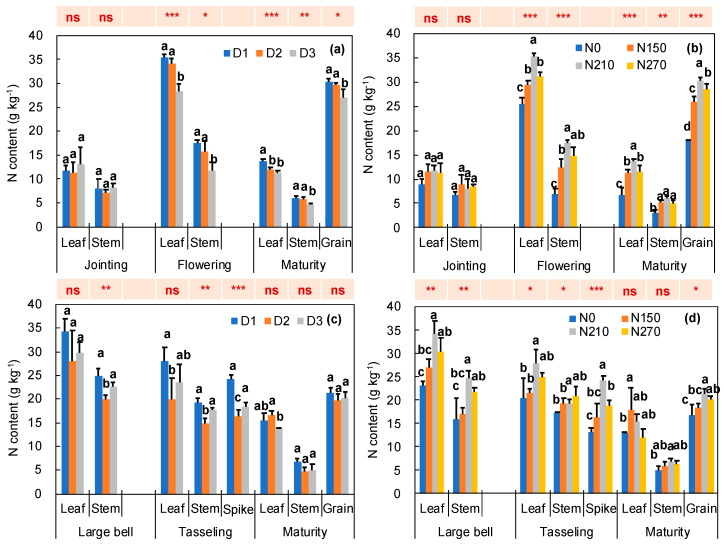
The effects of irrigation and fertilization on plant N contents in different parts of winter wheat (**a**,**b**) and summer maize (**c**,**d**) at different stages. Different lowercase letters indicate significant differences in the same growth stage at *p* < 0.05. The symbols of ns, *, **, and *** represent no significant effects and significant effects at 0.05, 0.01 and 0.001 significance levels of irrigation or fertilization in different growth stages. Definitions of different treatments (i.e., D1, D2, D3. N0, N150, N210 and N270) are given in caption of Table 1.

Irrigation significantly affected the N content in different parts of winter wheat at the flowering and maturity stages (Figure 2a). For example, at the maturity stage, the N content in leaves, stems, and grains was the highest in D1, with significant increase of 21.2%, 32.6%, and 12.2% compared with D3, respectively. Different N applications rates significantly affected the N content in different parts of winter wheat at the flowering and maturity stages (Figure 2b). Accordingly, the N210 treatment had the highest N content in leaves, stems, and grains at the maturity stage, reaching 13.7, 6.1, and 30.4 g kg^−1^, respectively.

In the summer maize season, irrigation significantly affected the stem N content at the large bell stage and the stem and ear N content at the tasseling stage, with the D1 treatment showing the highest values (Figure 2c). Different N applications rates also affected N content (Figure 2d). Moreover, the N210 treatment significantly increased the grain N content at the maturity stage (21.3 g kg^−1^), representing significant increase of 26.0% and 16.4% compared with N0 and N150, respectively.

### 3.3. Plant Enzyme Activity Characteristics

**Figure 3:**
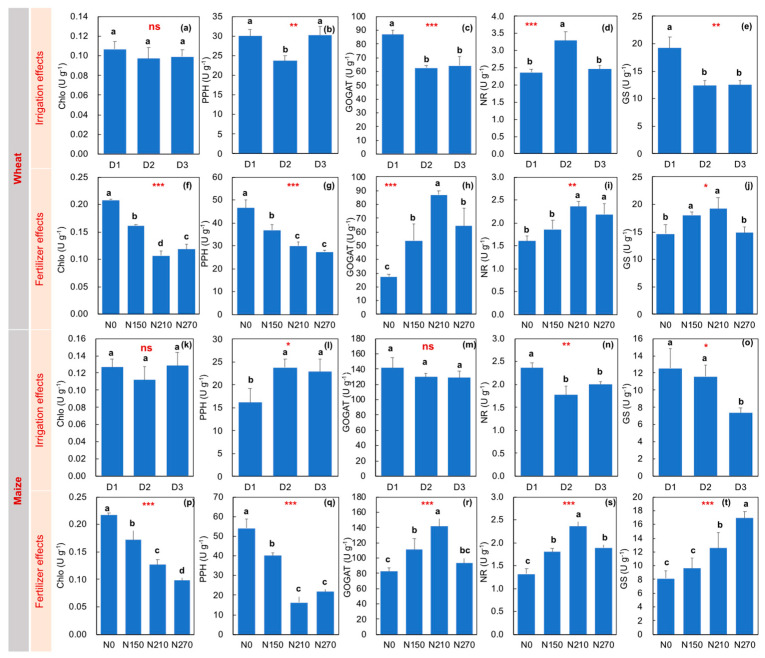
The effects of irrigation and fertilization on activities of chlorophyllase (Chlo), pheophytinase (PPH), glutamate synthase (GOGAT), nitrate reductase (NR), and glutamine synthetase (GS) of winter wheat (**a**–**j**) and summer maize (**k**–**t**) under different treatments. Different lowercase letters indicate significant differences at *p* < 0.05. The symbols of ns, *, **, and *** represent no significant effects and significant effects at 0.05, 0.01 and 0.001 significance levels of irrigation or fertilization. Definitions of different treatments (i.e., D1, D2, D3. N0, N150, N210 and N270) are given in caption of Table 1.

Irrigation significantly affected PPH and N-transforming enzyme activities in the winter wheat season (Figure 3). For the D2 treatment, PPH activity was 20.8% (*p* < 0.05) and 21.5% (*p* < 0.05) lower than those in D1 and D3, respectively, whereas NR activity was 39.8% (*p* < 0.05) and 33.6% (*p* < 0.05) higher, respectively. In addition, the Glutamate synthase (GOGAT) and Glutamine (GS) activities of D1 were significantly increased by 38.4%, 35.0% and 55.5%, 53.1% compared with D2 and D3, respectively. N application significantly affected chlorophyll content and N-transforming enzyme activities. Among all N treatments, N210 exhibited the lowest Chlo, while the activities of GOGAT, NR, and GS enzymes were the highest. In the summer maize season, irrigation amount significantly affected PPH, NR, and GS. The PPH activity of D1 was significantly decreased by 31.9% and 29.4% compared with D2 and D3, while NR and GS were significantly increased by 18.0% and 70.6% compared with D3. Similar to the winter wheat season, N application significantly affected chlorophyll content and N-transforming enzyme activities. PPH was the lowest in N210, and GOGAT and NR were the highest in N210. Overall, the D1 and N210 treatments exhibited the best plant physiological characteristics in both winter wheat and summer maize seasons (Figure S2).

### 3.4. Yield and N Utilization Characteristics

Irrigation amount significantly affected the N uptake of winter wheat at the flowering and maturity stages, with the D1 treatment showing the highest value, representing significant increase of 67.5% and 39.1% compared with D3, respectively (Table 1). Additionally, irrigation amount significantly affected summer maize yield, PFP, N uptake, and N translocation efficiency. Yield, PFP, and N uptake were all the highest in D1 treatment. Relative to D3, yield, PFP, and N uptake at both of flowering and maturity stages increased by 24.1%, 14.0%, and 25.7%, respectively.

Different N applications rates significantly affected winter wheat yield, PFP, and N uptake, as well as summer maize yield, PFP, N uptake, and N translocation efficiency. Across all treatments, the PFP, N uptake at flowering and maturity stages of winter wheat was the highest in the D1; the yield, N uptake at maturity stage, and BTPC of summer maize were the highest in N210.

### 3.5. Correlation Analysis

**Figure 4:**
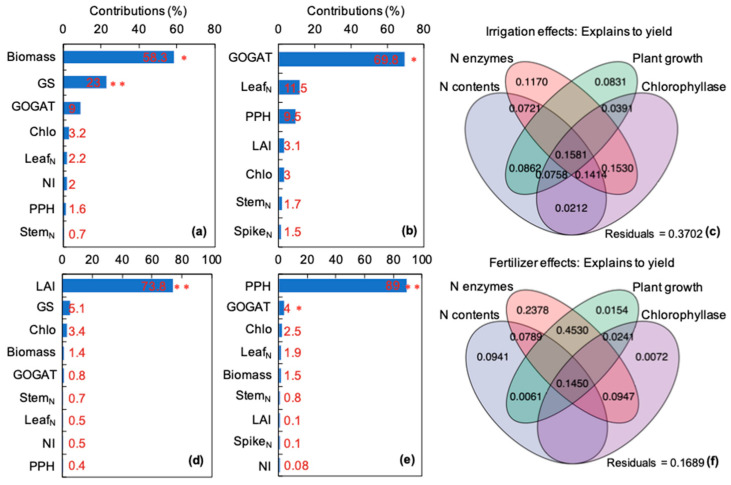
Redundancy analysis (RDA) and variance partitioning analysis (VPA) to reveal the yield-influencing factors. The effects of different plant growth factors to wheat yield (**a**), maize yield (**b**) and annual yield (**c**) under different irrigation treatments and the effects of different plant growth factors to wheat yield (**d**), maize yield (**e**) and annual yield (**f**) under different fertilization treatments. The symbols * and ** indicate that the growth factors of each plant under different irrigation or fertilization treatments have significant effects on the yield of wheat and maize at the 0.05 and 0.01 significance levels, respectively.

**Figure 5:**
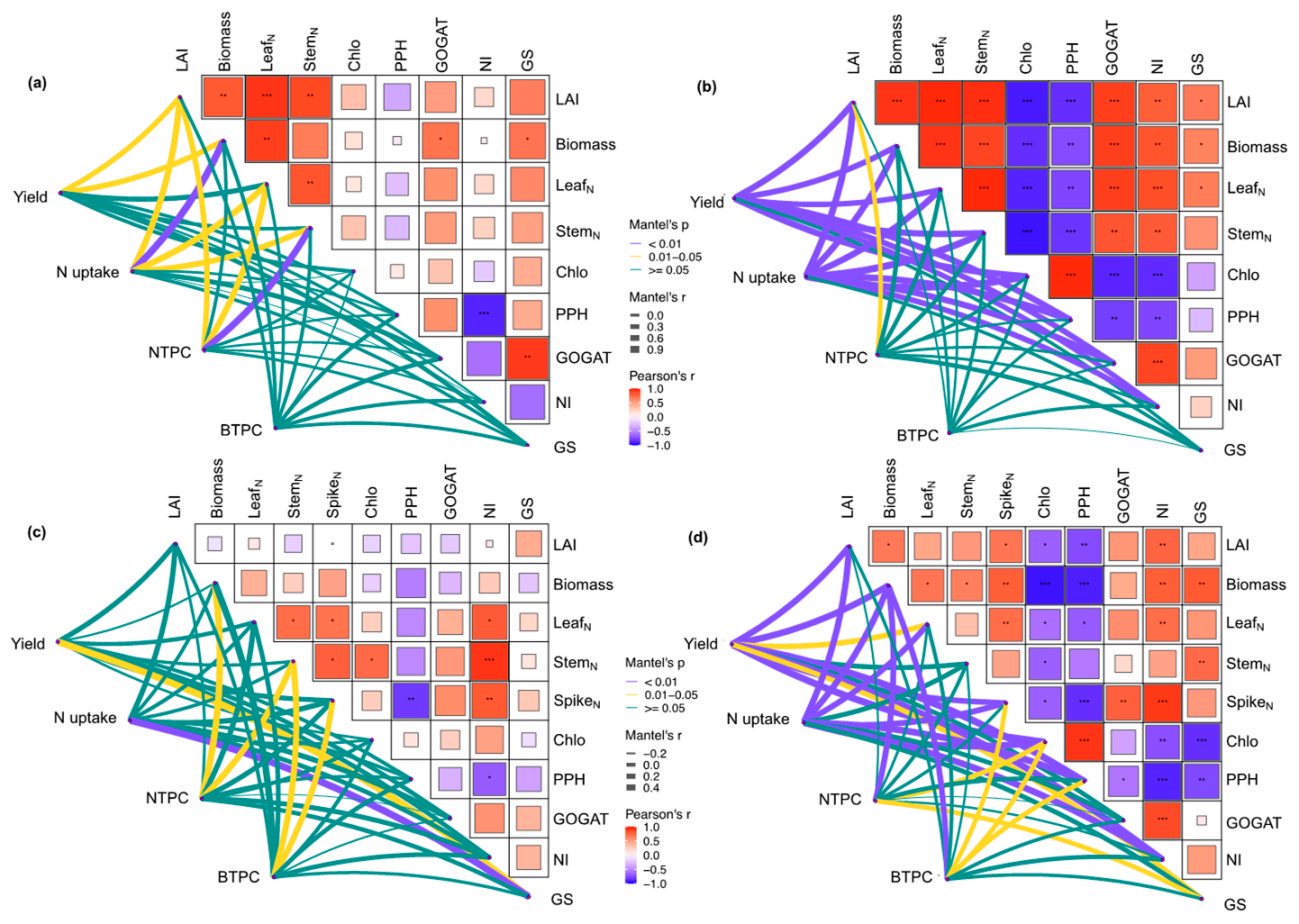
Correlation analysis between yield, PFP, N utilization characteristics and plant growth characteristics, N content, and enzymes of winter wheat (**a**,**b**) and summer maize (**c**,**d**) under different irrigation and N application conditions. The symbols *, ** and *** indicate that different irrigation or fertilization treatments have significant effects on the growth characteristics, plant N content and enzyme activities of wheat and maize at the 0.05, 0.01 and 0.001 significance levels, respectively.

Increasing irrigation amount and N application rate gradually increased the yields of winter wheat and summer maize (Figure S3). Under different irrigation treatments, biomass (*p* < 0.05), GS (*p* < 0.05), and GOGAT were the main factors affecting wheat yield (Figure 4a), while GOGAT (*p* < 0.05), leaf N content, and PPH were the main factors affecting maize yield under different irrigation treatments (Figure 4b). N uptake was significantly correlated with leaf and stem N content, and NTPC was significantly correlated with leaf and stem N content (Figure 5a). Under different N application treatments, LAI (*p* < 0.05) and GS were the main factors affecting wheat yield (Figure 4d), while PPH (*p* < 0.05) and GOGAT (*p* < 0.05) were the main factors affecting maize yield under different N application treatments (Figure 4e). N uptake was significantly correlated with LAI; NTPC, and BTPC was significantly correlated with stem N content (Figure 5c,d). According to the results of variance partitioning analysis (VPA), plant N-transforming enzymes and growth traits were the main factors affecting yield (Figure 4c,f).

**Figure 6:**
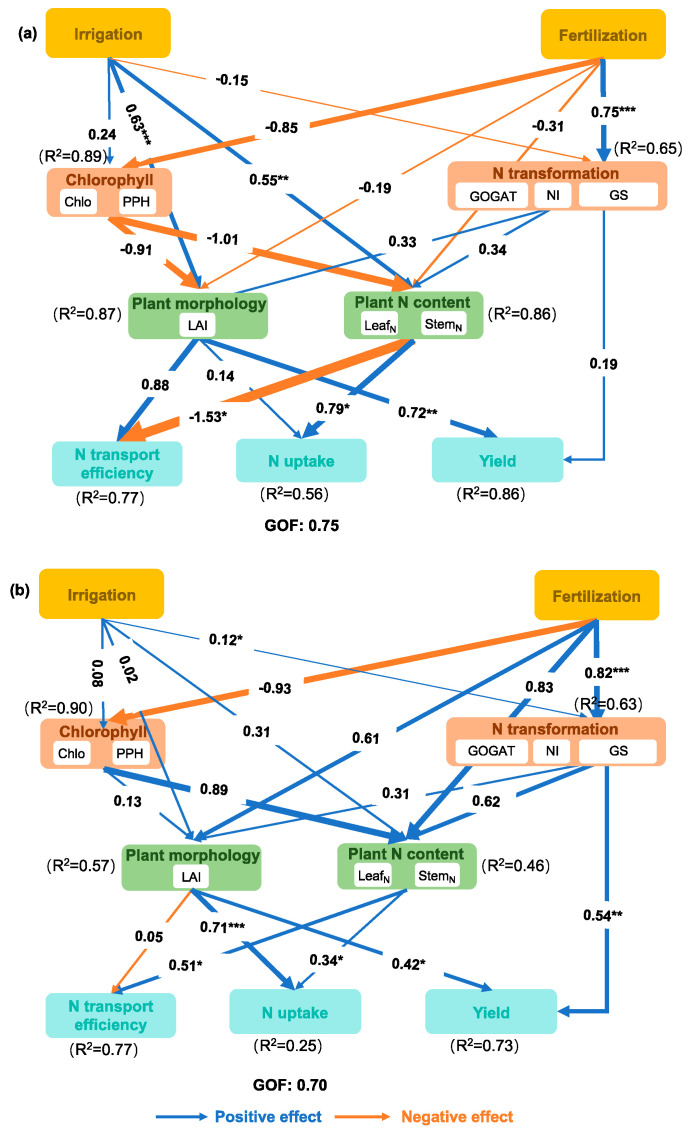
The structural equation model analysis of irrigation, fertilization plant growth characteristics, N content, and enzymes on N transport efficiency, uptake and yield in winter wheat (**a**) and summer maize (**b**) under different irrigation and fertilizer N treatments. The *, **, and *** represent significance levels of *p* < 0.05, 0.01, and 0.001, respectively.

Structural equation model analysis showed that chlorophyll-related enzymes, soil N cycle enzymes, leaf area index, and leaf–stem N content could directly regulate plant yield, nutrient translocation rate, and N uptake (Figure 6a,b). Among them, in the winter wheat season, irrigation promoted plant growth (via LAI) and increased yield, while fertilization directly and positively improved plant N-transforming enzyme activity (Figure 6a). In the summer maize season, both irrigation and fertilization significantly increased plant N-transforming enzyme activity, thereby improving yield (Figure 6b).

## 4. Discussion

### 4.1. Effects of Different Irrigation and N Management on Yield and N Utilization

In water-deficient areas (such as the North China Plain), reasonable water saving irrigation schemes were an important basis for alleviating the effects of water deficit during key growth periods, promoting plant (especially wheat) growth, and increasing yield [13]. As Zhou, et al. [14] confirmed that 66.7% of grain N originated from pre-flowering remobilization, while post-flowering uptake contributed the remaining 33.3%, which was consistent with the results of this study (Table 1). Under the same N application rate, moderate water stress was conducive to the translocation of N from leaves and stems to grains and enhanced the contribution rate of N translocation from vegetative organs to grains [15]. This study showed that as the irrigation amount increased from 52 mm (D3) to 127.8 mm (D1), the yields, PFP, N uptake, and NTPC of winter wheat and summer maize generally showed an upward trend (Table 1). However, in the summer maize season, the contribution rate of N translocation from vegetative organs to grains in the D3 treatment was higher than that of D1 and D2 treatments. A possible reason was that there was abundant rainfall during the late stage of maize flowering, which to some extent alleviated the water stress caused by the D3 irrigation deficiency. Based on the existing research findings [16], we speculated that moderate water deficiency supply may have stimulated root development and enhanced nitrogen uptake and transport capacity, which might be the potential reason for the observed results. Due to the limitations of the current observation indicators, this inference lacked the support of actual root physiological measurement data from this experiment. Further targeted experiments were needed to verify it.

Under water-deficient conditions, increasing N application could promote root growth and generate a large number of fine roots, thereby enhancing the plant’s ability to absorb nutrients and alleviating the reduction in N accumulation caused by water stress [17]. Under sufficient water supply, as N application rate increased, aboveground biomass and N use efficiency increased significantly; when the N application rate exceeds a certain threshold, the increase in N absorption efficiency and yield decreased significantly [18]. This study showed that there was a significant quantitative fitting relationship between annual yield and N application rate (Figure S3). Among them, the yields of winter wheat and summer maize were the highest in the N210 treatment, and the N uptake and the contribution rate of N translocation from vegetative organs to grains reached their peaks (Table 1). As Ju, et al. [19] reported that the optimal N application rate for wheat and maize in NCP was currently near 300 kg N ha^−1^ yr^−1^. Furthermore, under drip irrigation conditions, water–fertilizer coupling could further enable N to enter the effective root zone for crop absorption and utilization, thus having the potential to further reduce N application rate [20].

### 4.2. Effects of Different Irrigation and N Management on Crop Growth

Plant growth indicators (e.g., LAI, biomass) and N-transforming enzyme activities (e.g., GS, GOGAT) were the main factors affecting yield (Figure 4). Previous studies have shown that moderate irrigation could prevent anaerobic soil environment, facilitate root respiration, and improve N absorption and translocation efficiency [21]. Existing studies have shown that excessive nitrogen application promotes overgrowth of wheat leaves, reduces the leaf anti-senescence capacity, and impairs photosynthesis [22]. This study also found that in the fertilization treatment, GOGAT was the main factor affecting yield (Figure 4b), while compared with N210, N270 significantly reduced GOGAT (Figure 3h), thereby inhibiting the nitrogen transport in the aboveground plant tissues [23].Optimal N application could inhibit chlorophyllase activity, delay chlorophyll degradation, maintain photosynthetic efficiency [5], and enhance NR, GS, and GOGAT activities to promote plant N transformation, grain N accumulation and protein synthesis [6]. In our study, the D1 treatment combined with N210 treatment exhibited higher LAI, biomass, GS, and GOGAT enzyme activities at the flowering stage of winter wheat and the tasseling stage of maize, which resulted in the highest yield in all treatments (Figure 3).

Maize was considered as more sensitive to N fertilizer than wheat but had more stringent water requirements. The N270 treatment in the maize season had the largest LAI, while the D2 treatment had the highest biomass. The N270 treatment enhanced chlorophyll content and photosynthesis, yet excessive N decreased chlorophyllase activity (Figure 3), accelerating leaf senescence and reducing late-stage dry matter conversion [24]. Although the D2 treatment had slightly lower water and N inputs, it avoided the nutrient “dilution” effect caused by “excessive transpiration”, enhanced N-transforming enzyme activity, thereby enabling roots to efficiently convert absorbed N into amino acids and then into organic matter, which was preferentially supplied to grain development [25]. Therefore, only reasonable matching of water and N might help plant growth and improve the activity of key enzymes in the photosynthetic process, thereby achieving the goal of saving water, reducing N, and increasing yield.

### 4.3. Processes Underlying the Effects of Different Irrigation and N Management Practices on Crop Yield

Water and N management influence crop yield by modulating LAI, photosynthetic capacity and N assimilation, which collectively determine aboveground biomass and grain yield. Reduced activities of chlorophyllase (Chlo) and polyphenol oxidase (PPH) could delay leaf senescence and maintain a high photosynthetic rate for an extended period, leading to a significant increase in grain weight [24]. N cycle enzymes directly affect photosynthesis and N assimilation by regulating N formation and transformation, thus influencing crop yield and grain quality [11]. Mokhtari, et al. [26] identified LAI as the most reliable indicator of wheat yield, and a strong positive correlation between these two factors was observed in this study (Figure 5). In addition, the structural equation model analysis revealed that LAI and plant N content had a significant positive effect on the yield of both wheat and maize. For the maize growing season, irrigation and fertilizer N application improved grain yields by positively regulating plant N transformation processes (Figure 6b).

Existing research has demonstrated that drip irrigation could reduce irrigation water input by more than 50% compared with flood irrigation. This water saving practice not only inhibited the formation of anaerobic conditions in the rhizosphere soil but also imposed short-term water stress on crop roots, thereby enhancing plant tolerance to water stress through increasing in the activity of plant antioxidant enzymes, chlorophyll content, and photosynthetic performance [12]. These changes in crop physiological and morphological traits collectively improved photosynthetic efficiency and prolonged leaf longevity and promoted dry matter accumulation, ultimately contributing to higher grain yield.

However, the processes by which water and N management affect yield differed between wheat and maize (Figure 6). For the wheat growing season, water and N management regulated grain development primarily by modifying leaf photosynthate production and translocation [27]. In contrast, for the maize growing season, grain plumpness was determined through the regulation of root N uptake and transformation, especially the activity of key N-transforming enzymes [28]. These findings were further validated through the SEM analysis in this study. The dominant pathway of yield regulation in the maize growing season was through the modulation of plant N-transforming enzyme activity, which was due to the high N demand of maize—N is a key limiting factor for maize growth [29]. N-transforming enzymes (e.g., NR, GS, GOGAT) acted as the main regulators of N uptake, assimilation and translocation efficiency [23]. Therefore, irrigation and N application altered the activity of these N-transforming enzymes and further determined the final maize yield by regulating N assimilation and distribution in plants.

It should be noted that wheat and maize growth were controlled by multiple environmental factors, which restricts the extrapolation of our single-site experimental results under the unique climate of NCP; the optimized water–nitrogen regime proposed herein was only suitable for local analogous wheat–maize rotation system. Another key limitation was the non-full-factorial design, where irrigation and nitrogen managements were arranged separately, making it impossible to quantify their interaction effects. Future multi-site full factorial trials were therefore required to validate our findings. Subsequent studies could further clarify how water and nitrogen inputs shape root exudates, soil microbial communities and root nutrient absorption, and their linkages to the integrated soil N mineralization–plant N uptake–assimilation–utilization continuum.

## 5. Conclusions

The combination of D1 and 210 kg N ha^−1^ yr^−1^ represented the superior management regime in this region, which achieved high crop yields (wheat: 6.8–7.2 t ha^−1^; maize: 9.5–10.1 t ha^−1^) and improved N partial factor productivity (PFP, 42.3–45.7 kg kg^−1^). Correlation analysis indicated that this performance was accompanied by lower activities of chlorophyllase and PPH and higher activities of NR, GS and GOGAT, alongside higher LAI and aboveground biomass. RDA, VPA and SEM further revealed that growth traits and nitrogen metabolic enzymes were closely linked to yield formation. And the irrigation was mainly associated with yield variation via changes in LAI, whereas fertilization was correlated with yield through variations in nitrogen-metabolizing enzyme activity. This water and nitrogen regime could sustain high grain yield and high nutrient use efficiency, offering practical guidance for resource-conserving high-yield farming in the North China Plain.

## Data Availability

The data presented in this study are available on request from the corresponding author due to the ongoing nature of the research project, which involves further data collection and analysis that have not yet been completed.

## Supplementary Materials

The following supporting information can be downloaded at: https://www.mdpi.com/article/10.3390/plants15142171/s1.
