# Supplementary material for: Improving Crop Yield by Regulating Crop Growth and Nitrogen Transformation Through Water and Nitrogen Management Under Subsurface Drip Irrigation System
Source: Plants (Basel). 2026 Jul 15;15(14):2171. doi: 10.3390/plants15142171 (PMC13415087; doi:10.3390/plants15142171)
Supplement: Supplementary file 1 [file plants-15-02171-s001.zip › plants-4377957-supplementary.pdf]

Improving crop yield by regulating crop growth and nitrogen transformation through water and nitrogen management under subsurface drip irrigation system

Ziye Zhang[1], Yan Liu[1], Xin Zhang[1*], Aijun Zhang[1*], Yang Liu[1], Jing Zhou[1]

1 College of Resources and Environmental Sciences, Hebei Agricultural University, Baoding 071000, China

** Corresponding authors at: Hebei Agricultural University, Baoding, Hebei, China.*

E-mail: zhangxin_vic@hotmail.com (X. Zhang), zhangaijun@hebau.edu.cn (A. Zhang).

Experimental Design

Wheat sowing and drip tape laying were completed in one operation. The drip tapes were laid shallowly at an equal spacing of 60 cm, with a burial depth of 3~5 cm, for annual use. A split-plot design was adopted in the experiment.

For winter wheat, the base fertilizer was 90 kg N ha^{-1}, with two topdressings at the jointing and booting stages. For summer maize, the base fertilizers were 0 kg N ha^{-1}, 30 kg N ha^{-1}, 42 kg N ha^{-1}, and 54 kg N ha^{-1}, respectively, with two topdressings at the large bell and tasseling stages. If no supplementary irrigation was needed during the topdressing period, the minimum irrigation amount was used for fertilization. All treatments were applied with 135 kg ha^{-1} P_2O_5 and 105 kg ha^{-1} K_2O, and phosphorus and potassium fertilizers were applied once. Urea was used as topdressing. The irrigation system was equipped with pumps, filters, drip lines, electrovalves, and water meters. The drip lines were installed with a space of 60 cm in row. The pressure emitters had a water flow of 0.15 L h^{-1} and were spaced 30 cm apart. When the average of soil water content in 0 – 40 soil layer dropped below 65%, 60% and 55% field capacity, the depth (mm) of water applied was calculated using the following equation:

$$I=\frac{\rho p h \theta_f (q_1-q_2)}{\eta} \quad \text{(S1)}$$

where I represents the depth of irrigation water applied (mm); ρ represents the soil bulk density (1.34 g cm^{-3}); p represents the soil wetness ratio, 0.8; h represents the depth of the planned wetting soil layer (0.4 m); θ_f represents the maximum field water holding capacity and valued at 34.26%); q_1 and q_2 represent the irrigation upper limit (D1: 80%; D2: 75%; D3: 70%) and the measured soil moisture content (the ratio of the measured soil gravity water content to field capacity; %), respectively; and η represents the water use coefficient, which is assumed to be 0.95.

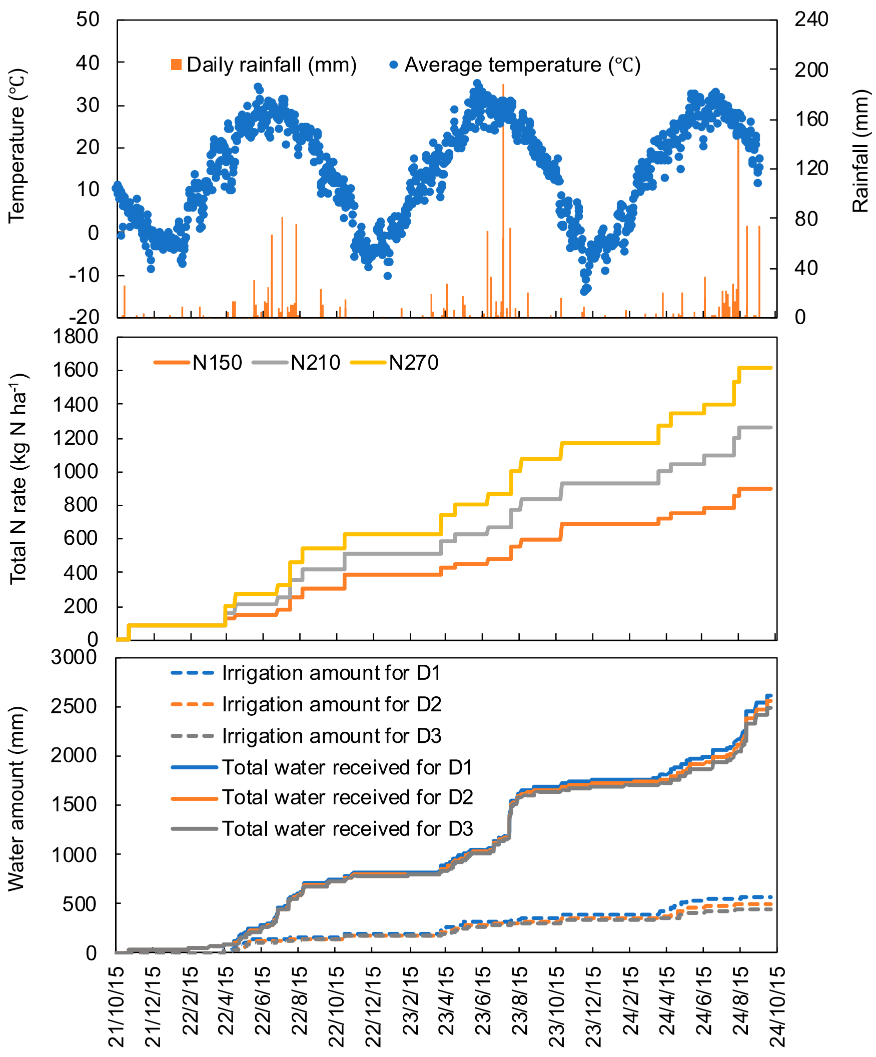

Figure S1 Temperature, rainfall, fertilization and irrigation amount during the experimental period. D1: irrigated to 80% of the field holding capacity when the soil water content was less than 65% of the field holding capacity; D2: irrigated to 75% of the field holding capacity when the soil water content was less than 60% of the field holding capacity; D3: irrigated to 70% of the field holding capacity when the soil water content was less than 55% of the field holding capacity, N0: no nitrogen application; N150: 150 kg N ha^{-1} per season; N210: 210 kg N ha^{-1} per season; and N270: 270 kg N ha^{-1} per season..

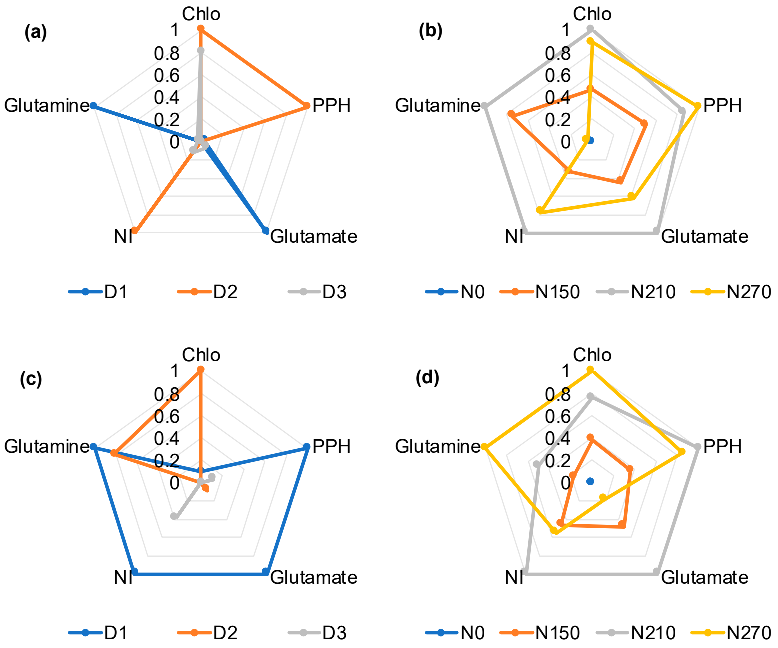

Figure S2 Radar chart of plant chlorophyllase and N-transforming enzymes. All data were standardized. Chlo and PPH were inversely standardized, that is, the lower the value, the stronger the leaf anti-aging ability. Definitions of different treatments (i.e., D1, D2, D3. N0, N150, N210 and N270) are given in caption of Table 1.

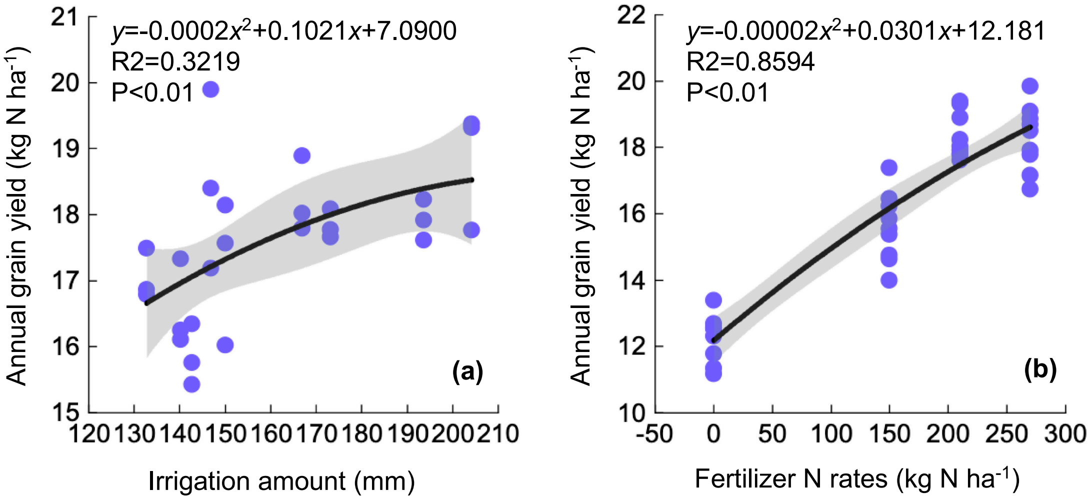

Figure S3 The correlationships between annual grain yields and irrigation amount under drip irrigation system (a) and fertilization (b) from 2022 to 2024.
